# Can CA-125 Predict Lymph Node Metastasis in Epithelial Ovarian Cancers in Turkish Population?

**DOI:** 10.1155/2014/492537

**Published:** 2014-03-25

**Authors:** Sinem Sudolmuş, Nadiye Köroğlu, Gökhan Yıldırım, Volkan Ülker, Ahmet Gülkılık, Ramazan Dansuk

**Affiliations:** ^1^Bezmialem Vakıf Üniversitesi, Adnan Menderes Bulvarı (Vatan Caddesi), Fatih, 34093 Istanbul, Turkey; ^2^Kanuni Sultan Suleyman Research and Teaching Hospital, Atakent Mh. 1. Cd, Küçükçekmece, 34303 Istanbul, Turkey

## Abstract

*Objective*. The role of single preoperative serum CA-125 levels in predicting pelvic or paraaortic lymph node metastasis in patients operated for epithelial ovarian cancer has been investigated. * Methods*. 176 patients diagnosed with epithelial ovarian carcinoma after staging laparotomy between January 2002 and May 2010 were evaluated retrospectively. * Results*. The mean, geometric mean, and median of preoperative serum CA-125 levels were 632,6, 200,29, and 191,5 U/mL, respectively. The cut-off value predicting lymph node metastases in the ROC curve was 71,92 U/mL, which is significant in logistic regression analysis (*P* = 0.005). The preoperative log CA-125 levels were also statistically significant in predicting lymph node metastasis in logistic regression analysis (*P* = 0.008). * Conclusions*. The tumor marker CA-125, which increases with grade independent of the effect of stage in EOC, is predictive of lymph node metastasis with a high rate of false positivity in Turkish population. The high false positive rate may obscure the predictive value of CA-125.

## 1. Introduction

Epithelial ovarian cancer (EOC) is the sixth most frequent cause of cancer death in women and is the leading cause of mortality resulting from overall gynecologic malignancies in the United States and Europe [1]. Over the world, there are approximately 224747 new cases of ovarian cancer per year resulting in more than 140163 deaths [1]. In Turkey, there are about 1804 new cases per year resulting in 1247 deaths [1].

Most patients with ovarian cancer have widespread disease at the time of diagnosis due to asymptomatic nature of early stage disease [2]. While the 5-year survival for women with International Federation of Gynecology and Obstetrics (FIGO) stage I or II (early stage disease) is approximately 90%, the 5-year survival of women diagnosed with FIGO stage III or stage IV (late stage disease) is less than 30% [3]. Furthermore, prevalence of pelvic or para-aortic lymph node metastasis is higher in EOC when compared with other gynecologic malignancies [4, 5]. Lymph node metastasis rate detected by systematic lymphadenectomy has been notified as 44% to 53% in unselected series containing all FIGO stages [6, 7].

Lymph node metastasis has been proven to be a prognostic factor in EOC [8, 9]. Lately, imaging studies, including computed tomography (CT), magnetic resonance imaging (MRI), and positron emission tomography (PET), are used to detect pelvic or para-aortic lymph node metastasis preoperatively. However, the prediction of lymph node metastasis by these modalities is not flawless and it is obvious that supplemental methods are required to designate pelvic or para-aortic lymph node metastasis preoperatively [8, 9].

Carbohydrate antigen 125 (CA-125) is a high molecular-weight glycoprotein expressed by epithelial ovarian tumors as well as on the surface of cells of mesothelial origin [10]. Currently, serum CA-125 levels, also accepted as a predictive and prognostic factor for ovarian cancers, are used for monitoring response after treatment and survival of patients. Although CA-125 in epithelial ovarian cancers, which varies extensively depending on racial and genetic differences, has been studied extensively, the effect of preoperative CA-125 levels in predicting lymph node metastases is controversial.

The purpose of the current study was to investigate single preoperative serum CA-125 level in predicting lymph node metastases in Turkish patients with EOC.

## 2. Material and Methods

The data of 225 patients who were diagnosed with EOC cancer after staging laparotomy at the Department of Gynecologic Oncology of Kanuni Sultan Suleyman Research and Teaching Hospital from January 2002 to May 2010 were retrospectively reviewed. Informed consent from the patients was not obtained because of the retrospective nature of the study. The study was approved by the ethics committee of the hospital (Approval no.: 249,12/06/2009).

The inclusion criteria were as follows: patients with histological confirmation of EOC; those who underwent pelvic or para-aortic lymphadenectomy during staging laparotomy; and those with preoperative imaging studies (CT and MRI) for the evaluation of lymph node involvement.

12 patients with endometrioid type EOC who had synchronous endometrioid type endometrial adenocarcinoma, 3 patients who also had tubal cancer, 5 patients who had metastases to ovaries from other genital organs or other organs, 2 patients who also had endometrial hyperplasia that can affect CA-125 levels, 8 patients operated for indications other than ovarian cancer and had no preoperative CA-125 levels or imaging studies, 10 patients who had staging laparotomy in another hospital and applied for secondary cytoreductive surgery, and 9 patients who were operated after neoadjuvant chemotherapy were excluded from the study. Finally there were 176 patients fulfilling the criteria for enrollment in the study.

Optimal debulking surgery was defined as a residual tumor ≤1 cm in maximal diameter. The tumors were staged according to the 2009 FIGO staging system and histologically defined according to World Health Organization (WHO) classification. All patients were referred to medical oncology for chemotherapy treatment after surgery.

Imaging studies were performed within one week before staging laparotomy. Preoperative serum CA-125 levels were measured before staging laparotomy using a radioimmunoassay kit (Roche F170 Moduler system). The upper normal value of serum CA-125 level was 35 U/mL. The cut-off value of single serum CA-125 level for the detecting of lymph node metastasis preoperatively was calculated using the receiver operating characteristic (ROC) curve and its significance was determined by logistic regression analysis with the odds ratio (OR) and its 95% confidence interval (CI). The logarithmic transformation of preoperative CA-125 (log CA-125) levels was also evaluated for predicting lymph node metastasis using logistic regression analysis (*P* = 0.008). Statistical analyses were performed using MedCalc statistical software (version 12—© 1993–2013 MedCalc Software bvba). A *P* value <0.05 was considered as statistical level of significance.

## 3. Results

**Figure 1:**
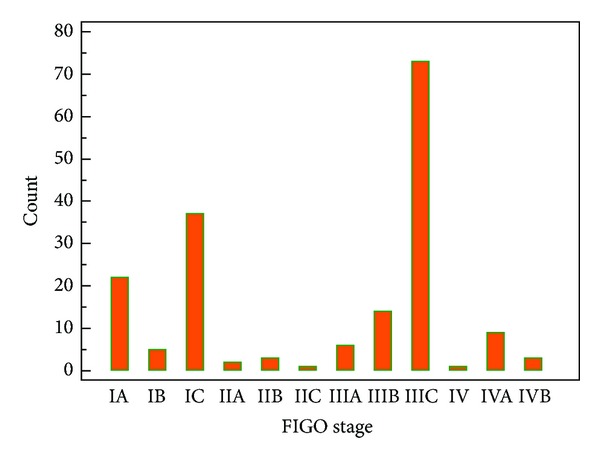
The FIGO stage distribution graph of patients.

The mean age of the 176 patients included in the study was 49,9 ± 12,2 while 89 of the cases (50,6%) were in menopausal state. The demographic features of the study population are shown in Table 1. The mean serum CA-125 of the patients was 632,6 U/mL while the values were in a very wide range: between 6,7 and 15554 U/mL. The geometric mean and median of preoperative serum CA-125 levels were 200,29 and 191,5 U/mL, respectively. The distribution of the patients among FIGO stages is shown in Figure 1. The most common FIGO stage among our patients was stage IIIC, with an incidence of 41,5% (73 patients).

**Figure 2:**
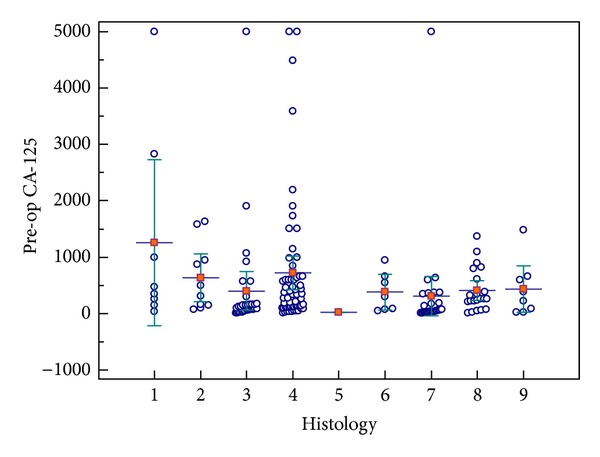
The distribution of histologic subtypes of epithelial ovarian cancers included in our study. The histologic types are as follows: (1) high grade clear cell; (2) high grade endometrioid; (3) high grade mucinous; (4) high grade serous; (5) low grade clear cell; (6) low grade endometrioid; (7) low grade mucinous; (8) low grade serous; (9) undifferentiated. (a) Bars for means, error bars for 95% CI for mean. (b) A case of high grade serous carcinoma with a CA-125 level of 15554 U/mL was excluded for graphic purposes.

Histologically, 46,6% of the tumors (82 cases) were serous cystadenocarcinoma, 34,1% (60 cases) were mucinous cystadenocarcinoma, 9,7% (17 cases) were endometrioid carcinoma, and 5,1% (9 cases) were clear cell carcinoma. 8 of the cases were diagnosed as undifferentiated tumors. The pathological specimens that could not be discerned if they are serous or mucinous were accepted as undifferentiated. The distribution of histologic subtypes of epithelial ovarian cancers in our study is shown in Figure 2.

In 47 cases (26,7%), lymph node metastasis was proven by pathological evaluation. Pelvic lymph node metastasis was observed in 35 patients (19,9%) and para-aortic lymph node metastasis was observed in 23 (13%). The mean number of positive lymph nodes acquired in pelvic or para-aortic lymphadenectomies was 7,8. The mean number of positive pelvic and para-aortic lymph nodes was 12,9 and 2,6, respectively.

When the cases were stratified for grade, the mean and median CA-125 levels in grade 1, grade 2, and grade 3 groups were 350,7 and 76; 526,7 and 140; and 641 and 239,5, respectively, and this distribution had a *P* = 0.0003. Two-way analysis of variance (ANOVA) showed that both grade and FIGO stage have significant effect on serum CA-125 levels, although grade's effect and stage's effect are not dependent on each other as shown by tests of between-subjects effects.

**Figure 3:**
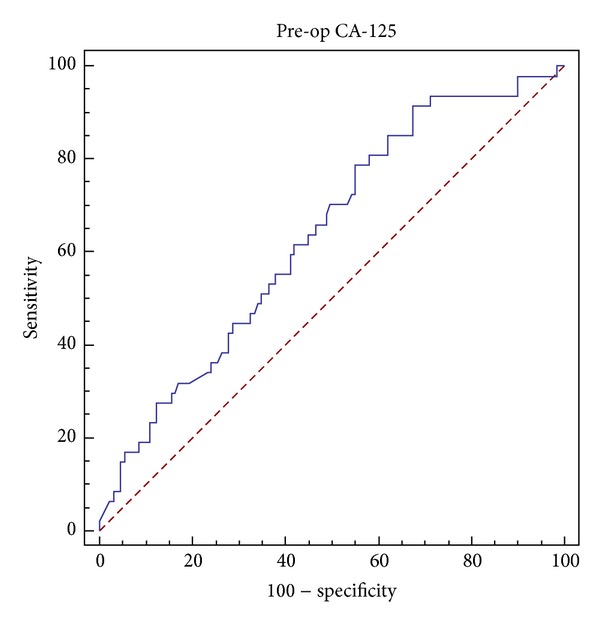
ROC curve for preoperative serum CA-125 levels.

The cut off value of serum CA-125 level in the ROC curve has been calculated by Youden index: max (Sensitivity + (Specificity – 100)). According to this equation, two optimal cut-off values with maximal Youden indices were further studied. The value with the highest index was 71,92 U/mL (area under curve (AUC) = 0,636 ± 0,05; *P* = 0.003) (Figure 3). This cut-off had a sensitivity of 91,49% (95% CI = 79,6–97,6%) and a specificity of 32,56% (95% CI = 24,6–41,4%). A cut-off value further from the initial cut-off in the coordinates of the ROC curve with a higher and clinically more acceptable specificity, also with a high Youden index, was 123 U/mL (sensitivity = 78,72%; 95% CI = 64,3–89,3% and specificity = 44,96%; 95% CI = 36,2–54%). When these levels were evaluated by logistic regression together with other factors shown in Tables 2 and 3, both were statistically significant for detecting lymph node metastasis in epithelial ovarian carcinoma (*P* = 0.005 and *P* = 0.013 for a CA-125 level of 72 and 123, resp.), and lymph node metastasis in imaging studies was found as a significant factor in detecting lymph node involvement. When log CA-125 as a continuous variable was evaluated for lymph node metastasis by logistic regression with the same clinical factors, it was also statistically significant (Table 4).

## 4. Discussion

While treating ovarian cancer, it is imperative to be prepared for an extended procedure preceding the operation. The tumor marker CA-125, the most extensively studied molecule for ovarian cancer in the literature, seems to be the most promising biomarker to predict the stage in a given patient [11–18]. We aimed to prove that a single preoperative serum CA-125 level can predict lymph node metastasis.

In our study, we found that a single preoperative serum CA-125 level can predict lymph node metastasis. However, the significant cut-off values we have ended up with are relatively low levels. For a serum CA-125 level of more than 72 and 123, the false positive ratio is 67,4% and 55%, respectively. While these values may be helpful in guiding clinical management, the false positive ratios are too high to use as a screening tool for predicting lymph node metastasis. Log CA-125 levels were also found statistically significant for predicting lymph node metastasis. On the other hand, logarithmic transformation of laboratory results in clinical settings is not always feasible. However, there are some studies that have shown preoperative CA-125 employable in detecting lymph node metastases. The study of Kim et al. on 99 Asian women with epithelial ovarian cancer has found the significant cut-off value of ≥535 U/mL which had a sensitivity and specificity in predicting lymph node metastasis similar to imaging studies [12]. Nonetheless, they could not succeed to prove that lymph node metastasis affects overall survival [12]. In another study they also stated that CA-125 levels are valuable in the prediction of advanced stage in patients who are diagnosed as early stage with CT and MRI [13]. Another similar study of the same group has found the significant CA-125 level to predict lymph node metastasis in endometrial cancer as 28 U/mL [14].

The cut-off value of 72 U/mL and the geometric mean serum CA-125 of 200,29 U/mL for all the patients enrolled in the study are relatively much lower than those values found in ovarian cancer studies mentioned above. The possible reasons of our lower values may stem from the fact that the number of early stage ovarian cancer patients is higher in our study population and a subset of patients who had lymph node metastasis and high CA-125 levels but were found eligible for neoadjuvant therapy were excluded during the enrollment process. However, serum CA-125 levels may rise above normal because of the tumor extension and metastasis while tumor mass is another overlapping source of high serum CA-125 levels. Namely, high serum CA-125 levels that may relate to lymph node metastasis may also be encountered in women with advanced stage disease. Our exclusion of advanced stage cases was supposed to eliminate this effect; meanwhile it lowered our CA-125 values in general. However, it has also been shown that peritoneal implants outside the pelvis may occur in lower serum CA-125 values than values observed in patients with retroperitoneal lymph node metastasis [13].

In a recent meta-analysis of imaging studies in the detection of lymph node involvement in ovarian cancer by Yuan et al., CT and MRI did not have optimal performance in detecting lymph node metastasis (sensitivity: 42.6% and 54.7%; specificity: 95.0% and 88.3%; OR: 19.87 and 12.38 for CT and MRI, resp.) while PET or PET/CT was a more accurate modality (sensitivity: 73.2%; specificity: 96.7%; OR [odds ratio]: 90.32) [15]. Although we found that preoperative single serum CA-125 levels are significant, clinical use of the low serum CA-125 levels statistically calculated for predicting lymph node metastasis in our study may lead to overdiagnosis. Serum CA-125 levels were not a better means than imaging studies to predict lymph node metastasis in our study, in which we did not use the more accurate studies of PET or PET/CT.

In our study, serum CA-125 levels, behaving typically as other tumor markers, increased gradually with increasing grade of the lesions independent of the effect of the stage. This was shown to be statistically significant, as seen in the literature [17].

Many tumor markers have been studied for screening and tumor progression in ovarian cancer. The cancer cell or the immune response against the tumor through the extracellular matrix, the vasculature, and the patient's body fluids has been investigated thoroughly with many approaches from transcripts to posttranslational modifications, producing ample markers for research in the diagnosis, screening, and prognosis of ovarian cancer [18, 19]. However, despite extensive research, no single tumor marker seems to overcome CA-125 [18]. Moreover, CA-125 alone appears superior to panels developed for detection of ovarian cancer in many studies, yet human epididymis protein 4 (HE4) has been offered to have superior specificity in laboratory identification of ovarian cancer in suspected gynecological lesions [20–22].

Race, irregular periods, oral contraceptive use, bilateral oophorectomy, and history of infertility treatment were also reported to be altering baseline CA-125 levels, suggesting CA-125 cut-off could be tailored for individuals in the future if prospective studies to be conducted support these findings [16]. CA-125 levels are above normal limits in 1% of healthy blood donors, in 29% of other cancers (lung, breast, pancreas, and colorectum), and in 6% of women with nonmalignant conditions (cirrhosis with ascites, acute pancreatitis, ovarian cysts, endometriosis, and pelvic inflammatory disease) [23], and it is almost impossible to eliminate factors increasing CA-125 to unify study populations for a clear-cut evaluation, despite the fact that we have excluded racial differences in our present study. Therefore, there is no clear explicit consensus on the use of CA-125 in screening, disease progression, and follow-up in ovarian cancer, letting alone other less intensively investigated tumor markers. Nevertheless, CA-125 is used as a sound marker to predict malignancy in pelvic masses [24]. There are also many studies evaluating the role of CA-125 in predicting prognosis in ovarian cancer with the context of different methods, for example, area under curve, nadir, and longitudinal monitoring of CA-125 levels [25–28].

There are some limitations present in our study, which stem from the retrospective nature of the design of the study, relatively small number of subjects, and the uneven distribution of ovarian cancer stages among these subjects that may obscure the statistical significance of CA-125. Furthermore, solely a single preoperative value of CA-125 level was investigated while the rate of increase of CA-125 preoperatively or comparison of preoperative levels with baseline levels may be analyzed for further research in predicting lymph node metastasis. Postoperative serum CA-125 levels have also been demonstrated as prognostic factors in ovarian cancer [25–28].

In conclusion, many physiologic processes and racial and genetic variations may contribute to the versatility of the tumor marker CA-125, which increases with grade independent of the effect of stage in EOC and is predictive of lymph node metastasis with a high rate of false positivity in Turkish population. The high false positive rate may obscure the predictive value of CA-125 and the role of CA-125 in this issue needs to be settled. Further studies on preoperative CA-125 provide a vast area to be explored in detecting lymph node metastasis or advanced stage disease in EOC.

## Figures and Tables

**Table 1:** Demographic features of patients enrolled in the study.

	*N*	Mean	95% CI	SD	Median	Minimum	Maximum	25–75 percentile
Age	176	49,9	48,1–51,7	12,2	49	18	84	42,5–58,5
Preoperative CA-125	176	632,6	410,3–854,9	1494,4	191,5	6,7	15554	69,7–600
Gravidity	176	3,6	3,2–4	2,7	3	0	13	2–5
Parity	176	2,8	2,5–3,1	2,2	2	0	13	2-3
Weight	176	69,8	68,1–71,5	11,3	68	45	115	62,5–76
Age of menarche	176	13,5	13,3–13,7	1,5	13	10	18	13-14

**Table 2:** Logistic regression analysis of 72 as a cut-off for CA-125 and independent clinical factors in predicting lymph node metastasis.

Variable	*β*	Standard error	*P*	Odds ratio	95% confidence interval
Age (quantitative)	0,029	0,016	0.067	0,971	0,942 to 1,002
Histology serous or nonserous	0,166	0,191	0.383	1,181	0,813 to 1,716
Preoperative CA-125 > 72 U/mL	0,808	0,290	**0.005**	2,242	1,271 to 3,955
Lymph node metastasis in imaging studies	1,131	0,413	**0.006**	3,100	1,379 to 6,970

Model *χ* ^2^ = 26,23; *P* < 0.001; overall classification 77,3%.

**Table 3:** Logistic regression analysis of 123 as a cut-off for CA-125 and independent clinical factors in predicting lymph node metastasis.

Variable	*β*	Standard error	*P*	Odds ratio	95% confidence interval
Age (quantitative)	−0,022	0,016	0.157	0,978	0,949 to 1,009
Histology serous or nonserous	0,123	0,189	0.515	1,131	0,781 to 1,638
Preoperative CA-125 > 123 U/mL	0,526	0,211	**0.013**	1,692	1,119 to 2,559
Lymph node metastasis in imaging studies	1,250	0,421	**0.003**	3,492	1,531 to 7,964

Model *χ* ^2^ = 22,83; *P* < 0.001; overall classification 76,7%.

**Table 4:** Logistic regression analysis of log CA-125 and independent clinical factors in predicting lymph node metastasis.

Variable	*β*	Standard error	*P*	Odds ratio	95% confidence interval
Age (quantitative)	0,028	0,016	0.073	0,972	0,943 to 1,003
Histology serous or nonserous	0,149	0,191	0.435	1,161	0,798 to 1,689
Preoperative log CA-125	0,345	0,129	**0.008**	1,412	1,096 to 1,818
Lymph node metastasis in imaging studies	1,206	0,415	**0.004**	3,338	1,481 to 7,525

Model *χ* ^2^ = 23,49; *P* < 0.001; overall classification 77,3%.

## References

[1] (2010). *GLOBOCAN, 2008, Cancer Incidence and Mortality Worldwide*.

[2] Jemal A, Thomas A, Murray T, Thun M (2002). Cancer statistics, 2002. *Ca: A Cancer Journal for Clinicians*.

[3] Fishman DA, Bozorgi K (2002). The scientific basis of early detection of epithelial ovarian cancer: the National Ovarian Cancer Early Detection Program (NOCEDP). *Cancer Treatment and Research*.

[4] Pecorelli S, Benedet JL, Creasman WT, Shepherd JH (1999). FIGO staging of gynecologic cancer. *International Journal of Gynecology and Obstetrics*.

[5] Onda T, Yoshikawa H, Yasugi T (1998). Patients with ovarian carcinoma upstaged to stage III after systematic lymphadenctomy have similar survival to Stage I/II patients and superior survival to other Stage III patients. *Cancer*.

[6] Harter P, Gnauert K, Hils R (2007). Pattern and clinical predictors of lymph node metastases in epithelial ovarian cancer. *International Journal of Gynecological Cancer*.

[7] Morice P, Joulie F, Camatte S (2003). Lymph node involvement in epithelial ovarian cancer: analysis of 276 pelvic and paraaortic lymphadenectomies and surgical implications. *Journal of the American College of Surgeons*.

[8] di Re F, Baiocchi G, Fontanelli R (1996). Systematic pelvic and paraaortic lymphadenectomy for advanced ovarian cancer: prognostic significance of node metastases. *Gynecologic Oncology*.

[9] di Re F, Baiocchi G (2000). Value of lymph node assessment in ovarian cancer: status of the art at the end of the second millennium. *International Journal of Gynecological Cancer*.

[10] Jacobs I, Bast RC (1989). The CA 125 tumour-associated antigen: a review of the literature. *Human Reproduction*.

[11] Pectasides D, Fountzilas G, Aravantinos G (2007). Epithelial ovarian carcinoma in younger vs older women: is age an independent prognostic factor? The Hellenic Oncology Cooperative Group experience. *International Journal of Gynecological Cancer*.

[12] Kim HS, Park NH, Chung HH, Kim JW, Song YS, Kang SB (2008). Significance of preoperative serum CA-125 levels in the prediction of lymph node metastasis in epithelial ovarian cancer. *Acta Obstetricia et Gynecologica Scandinavica*.

[13] Kim HS, Kim JW, Cho JY (2009). The role of serum CA-125 levels in early-stage epithelial ovarian cancer on preoperative CT and MRI. *European Journal of Surgical Oncology*.

[14] Hoon Chung H, Weon Kim J, Park N-H, Song Y-S, Kang S-B, Lee H-P (2006). Use of preoperative serum CA-125 levels for prediction of lymph node metastasis and prognosis in endometrial cancer. *Acta Obstetricia et Gynecologica Scandinavica*.

[15] Yuan Y, Gu Z-X, Tao X-F, Liu S-Y (2012). Computer tomography, magnetic resonance imaging, and positron emission tomography or positron emission tomography/computer tomography for detection of metastatic lymph nodes in patients with ovarian cancer: a meta-analysis. *European Journal of Radiology*.

[16] Skates SJ, Mai P, Horick NK (2011). Large prospective study of ovarian cancer screening in high-risk women: CA125 cut-point defined by menopausal status. *Cancer Prevention Research*.

[17] Cooper BC, Sood AK, Davis CS (2002). Preoperative CA 125 levels: an independent prognostic factor for epithelial ovarian cancer. *Obstetrics and Gynecology*.

[18] Sasaroli D, Coukos G, Scholler N (2009). Beyond CA125: the coming of age of ovarian cancer biomarkers. *Biomarkers in Medicine*.

[19] Gnjatic S, Ritter E, Büchler MW (2010). Seromic profiling of ovarian and pancreatic cancer. *Proceedings of the National Academy of Sciences of the United States of America*.

[20] Zhu CS, Pinsky PF, Cramer DW (2011). A framework for evaluating biomarkers for early detection: validation of biomarker panels for ovarian cancer. *Cancer Prevention Research*.

[21] Cramer DW, Bast RC, Berg CD (2011). Ovarian cancer biomarker performance in prostate, lung, colorectal, and ovarian cancer screening trial specimens. *Cancer Prevention Research*.

[22] Ferraro S, Braga F, Lanzoni M, Boracchi P, Biganzoli EM, Panteghini M (2013). Serum human epididymis protein 4 vs carbohydrate antigen 125 for ovarian cancer diagnosis: a systematic review. *Journal of Clinical Pathology*.

[23] Scholler N, Urban N (2007). CA125 in ovarian cancer. *Biomarkers in Medicine*.

[24] Medeiros LR, Rosa DD, da Rosa MI, Bozzetti MC (2009). Accuracy of CA 125 in the diagnosis of ovarian tumors: a quantitative systematic review. *The European Journal of Obstetrics & Gynecology and Reproductive Biology*.

[25] Uzunoglu S, Aybatlı A, Kaplan PB (2013). Assessment of CA-125 area under the curve as a prognostic factor in patients with ovarian cancer. *Medical Oncology*.

[26] Xu X, Wang Y, Wang F (2013). Nadir CA-125 level as prognosis indicator of high-grade serous ovarian cancer. *Journal of Ovarian Research*.

[27] Chen X, Zhang J, Cheng W (2013). CA-125 level as a prognostic indicator in type I and type II epithelial ovarian cancer. *International Journal of Gynecological Cancer*.

[28] Gupta D, Lammersfeld CA, Vashi PG, Braun DP (2010). Longitudinal monitoring of CA125 levels provides additional information about survival in ovarian cancer. *Journal of Ovarian Research*.

